# Characterization of Polyhydroxybutyrate-Based Composites Prepared by Injection Molding

**DOI:** 10.3390/polym13244444

**Published:** 2021-12-18

**Authors:** Marcos M. Hernandez, Nevin S. Gupta, Kwan-Soo Lee, Aaron C. Pital, Babetta L. Marrone, Carl N. Iverson, Joseph H. Dumont

**Affiliations:** C-CDE Chemical Diagnostics and Engineering, Los Alamos National Laboratory, Los Alamos, NM 87545, USA; mhernandez@lanl.gov (M.M.H.); nevin5@lanl.gov (N.S.G.); kslee@lanl.gov (K.-S.L.); acpital@lanl.gov (A.C.P.); blm@lanl.gov (B.L.M.); iverson@lanl.gov (C.N.I.)

**Keywords:** biodegradable plastics, polyhydroxybutyrate, polymer composites, compounding, injection molding

## Abstract

The waste generated by single-use plastics is often non-recyclable and non-biodegradable, inevitably ending up in our landfills, ecosystems, and food chain. Through the introduction of biodegradable polymers as substitutes for common plastics, we can decrease our impact on the planet. In this study, we evaluate the changes in mechanical and thermal properties of polyhydroxybutyrate-based composites with various additives: Microspheres, carbon fibers or polyethylene glycol (2000, 10,000, and 20,000 MW). The mixtures were injection molded using an in-house mold attached to a commercial extruder. The resulting samples were characterized using microscopy and a series of spectroscopic, thermal, and mechanical techniques. We have shown that the addition of carbon fibers and microspheres had minimal impact on thermal stability, whereas polyethylene glycol showed slight improvements at higher molecular weights. All of the composite samples showed a decrease in hardness and compressibility. The findings described in this study will improve our understanding of polyhydroxybutyrate-based composites prepared by injection molding, enabling advancements in integrating biodegradable plastics into everyday products.

## 1. Introduction

Single use plastics (SUPs) are fossil fuel-based materials commonly used in the food and beverage industry, designed to be disposed of immediately after use. These plastics, often composed of polypropylene, polystyrene, or polyethylene are historically challenging to recycle, and current waste collection systems do not have the capacity to safely and effectively dispose of our recycle waste plastic on a global scale [1]. As a result, these SUPs that end up in landfills slowly make their way into our ecosystems, oceans, and our food chain, contributing to society’s growing plastic pollution problems [2,3,4,5]. Current forecasts show that by 2050, only 26% of the 33 billion tons of plastics produced worldwide will have been recycled. To reduce the alarming pollution caused by our plastics consumption, the development and transition to truly biodegradable plastics is crucial.

Polyhydroxybutyrates (PHBs) are polyesters, which can be biologically produced by microorganisms, such as cyanobacteria or synthetically produced [6,7]. While these materials were first discovered in the 1920s, they only recently have gained interest due to their biodegradable nature [8]. In fact, recent studies have shown that PHB anaerobically degrades more rapidly at moderate temperatures than other biomaterials [9]. PHBs are used in industries, such as biomedicine and agriculture [10,11,12]. Although PHBs are extremely attractive for their degradability, they are also known for possessing limited mechanical properties due to the presence of large crystals in the material, making it unsuitable for applications, such as flexible packaging films [13]. Furthermore, the manufacture of these materials using industrial film/packaging equipment is rendered more difficult by the proximity between their degradation and melting temperatures [14,15,16]. While efforts have been made to predict the material thermal behavior and find PHBs suitable for a wide range of applications, more experimental work and modifications are needed to improve the performance of the materials [17,18]. 

Compounding, i.e., incorporation of additives into polymers, is essential to the manufacturing of polymer composites and the modification of their chemical, thermal, and mechanical properties. For instance, carbon fibers have been used to improve the thermomechanical and electrical properties of polymers, as well as to strengthen the material [19,20,21,22]. Microspheres are hollow fillers that are often used as a blowing agent to reduce the density of a variety of materials and improve their compressibility with applications ranging from footwear to the space industry [23,24,25]. Finally, polyethylene glycol (PEG) is a biodegradable polyether, used for its plasticizing properties to decrease polymer rigidity, which increases the materials process ability, as well as to provide lubricating coatings and decrease brittleness [26,27].

While carbon fibers, microspheres, and PEG in other polymers have been extensively described in the literature, there is little to no information on their effects when incorporated in PHB. In fact, the thermal and mechanical properties of PHB composites have not been well explored and this study is an attempt to fill the gap in the literature. Here, we compound these additives with PHBs, using a single-screw extruder retrofitted with an in-house injection mold. The injection-molded samples are an attempt to produce parts using a state-of-the-art industrial technique to modify the mechanical properties without altering the intrinsic nature of the materials. The samples were characterized using a wide array of microscopy, spectroscopic, thermal, and mechanical techniques, such as Fourier-Transform InfraRed (FT-IR) spectroscopy, thermogravimetric analysis (TGA), hardness (Shore A), and compression testing. 

Improving the chemical, thermal, and mechanical properties of PHBs, while retaining their biodegradable nature is essential to transition from petroleum-based to biodegradable plastics and reduce the impact of plastic pollution around the world. This work will help identify the additives and processing conditions for PHB biopolymers and composites that will accelerate their wider adoption.

## 2. Materials and Methods

### 2.1. Materials

PHB granules (BU396312) were purchased from Goodfellow Cambridge Ltd. located in Cambridge, United Kingdom. Carbon fibers (Pyrograff PR-19-XT-PS) were purchased from Applied Sciences, United States. Expancel 930DU120 microspheres were purchased from Nouryon Company located in Bohus Ale, Sweden. The 2000 molecular weight (MW) PEG was purchased from Alfa Aesar, located in Tewksbury, Massachusetts, USA. Ten thousand and 20,000 MW PEG were purchased from Sigma-Aldrich, located in St. Louis, MO, USA. An EX2 model extruder was purchased from Filabot (Barre, VT, USA). All of the materials were used as received.

### 2.2. Methods

**Figure 1:**
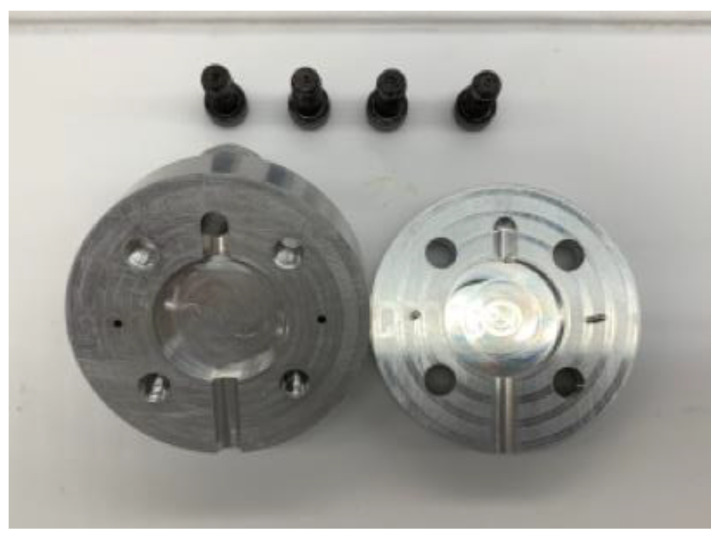
Custom split-die injection mold with a cavity size of 25.4 × 6.35 mm used to form samples for compression and hardness testing.

Injection Molding: The EX2 was preheated to 200 °C and fitted with an injection mold. The EX2 is a single-screw extruder that is equipped with a compact 3 stage extrusion screw. The stainless steel screw is proprietary to Filabot, and designed to pressurize the material during processing. The heating element in the extruder, allows for a relatively consistent temperature along the extrusion screw. A custom injection mold was designed and manufactured to produce a 6.35 mm thickness disk at 25.4 mm diameter (Figure 1). This geometry was selected for its repeatability and ease of use in mechanical testing. The custom mold was attached at the nozzle end of the extruder and allowed to equilibrate with the preheated extruder. For sample production, the rate of extrusion was approximately 75% (18 RPM) of the full capability.

Samples were prepared by combining additives with PHB granules (*w*/*w*) in a zip lock bag and mixed by shaking. After the contents appeared to be well mixed, they were poured into the hopper, and extruded until the material exited the outlet of the injection mold. Then, the extruder was shut off, and the material was held at room temperature for 1 min in the mold. Thereafter, the mold was removed from the extruder and cooled in a water bath prior to sample removal.

The density was estimated by taking the sample mass (obtained by scale) and dividing it by the volume of the sample.

**Figure 2:**
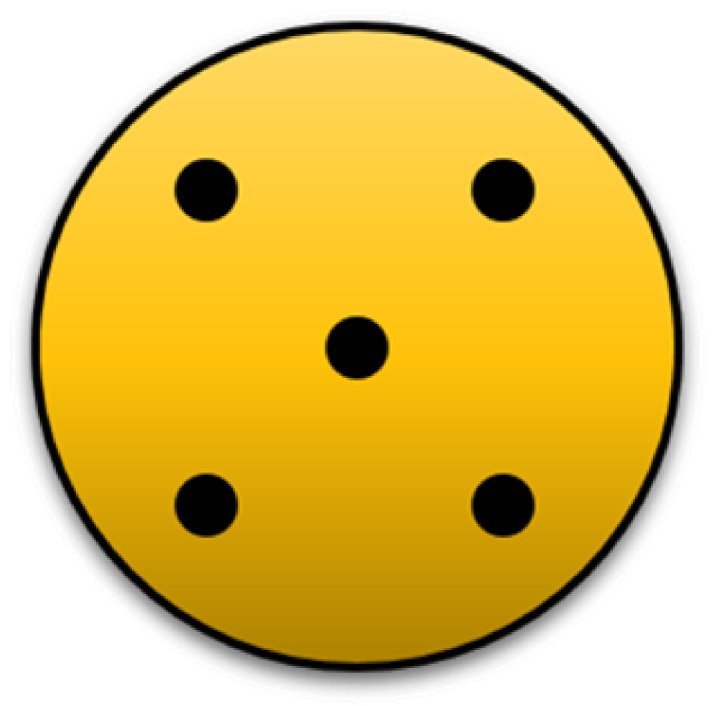
Shore A hardness probing sites along the sample surface.

Hardness testing: A Shore A digital hardness probe and test stand manufactured by Zwick Roell Group (Ulm, Germany) was used to test the surface hardness of the discs. Each disc was probed at five different locations on the surface. The five probing sites were chosen uniformly across all of the samples as shown in Figure 2. Then, the collected values were averaged to find the hardness of each sample.

**Figure 3:**
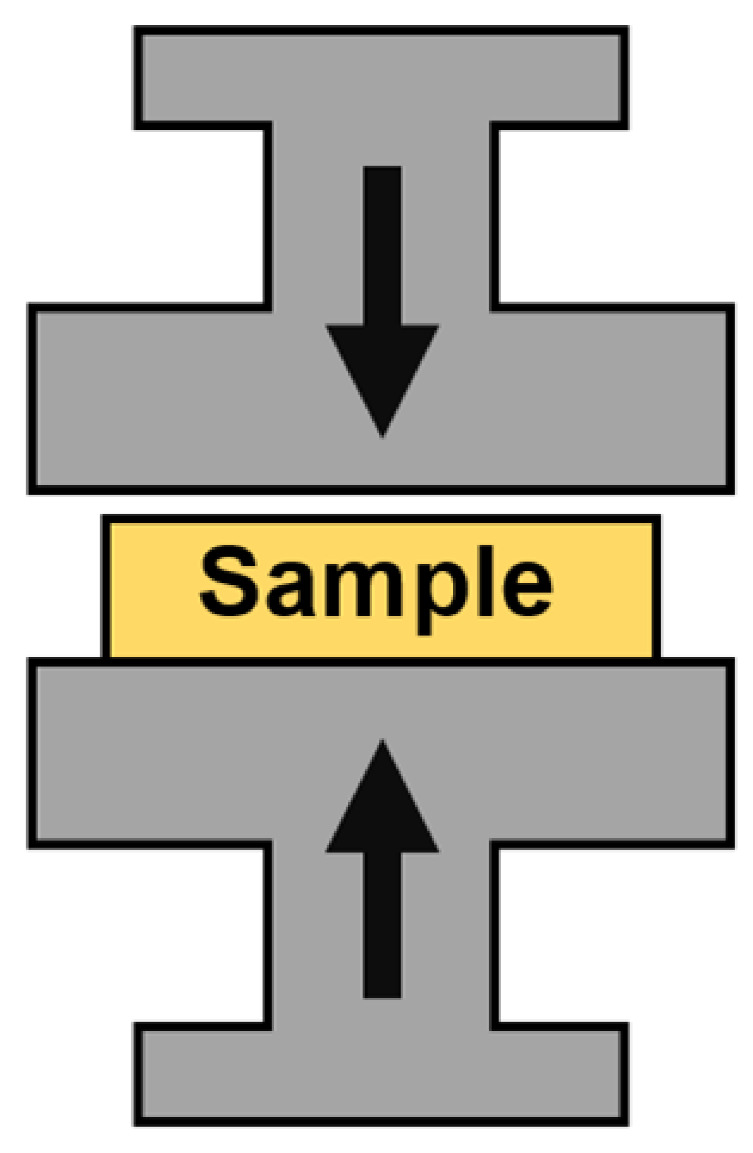
Compression testing configuration.

Compression testing: A 3300 series single column testing system produced by Instron Co. (Norwood, MA USA) was used to test the compressibility of the discs. Instrom Co. compression platens (model 2501-083) were used as the compression attachment (refer to Figure 3. Samples were compressed to 1 MPa at a rate of 0.02 mm/s. Four compression cycles were completed, with the third cycle used to analyze the percent deformation due to the compression for a comparison between the formulations. This deformation is representative of the change in dimension of the sample thickness due to the compressibility of the material.

Fourier Transform Infrared (FT-IR) spectroscopy: A Nicolet iS50 FT-IR manufactured by Thermo Fisher Scientific (Waltham, MA USA), was used to analyze the chemical compounds present in the samples. Thirty-two scans were collected for each sample, with the background collected after every 120 min. 

Thermogravimetric Analysis (TGA): A TGA 550 produced by TA Instruments (New Castle, DE, USA) was used to analyze the thermal degradation (T_d_) of the samples. All of the tests were performed in a nitrogen filled test chamber. The following method was used: Equilibrate to 50 °C, heat 10 °C/min to 900 °C, end cycle. Trios software (TA instruments) was used to analyze the data. T_d_ was reported at the 5% wt loss across all of the samples.

Microscopy: A VHX 6000 digital microscope from KEYENCE (Osaka, Japan) was used to characterize the surface conditions of the samples. Images were taken at 20× and 100×, with full ring lighting. For microsphere composites, a partial ring was used to accent the pores present in the sample. Photographs and microscopy images were taken to evaluate the visual changes of the prepared samples and analyze changes in porosity, discoloration or surface imperfections. Cross sections were taken by cutting an outlet filament from the mold exit in half.

Scanning Electron Microscopy: Analysis of the sample morphology was performed using a FEI Quanta 200 Scanning Electron Microscope (SEM). PHB samples were sliced and mounted with carbon tape on standard ½” SEM posts. Samples were Au coated to an approximate thickness of (0.2 mA for 60 seconds), prior to testing to avoid charging on the surface, since the sample is non-conductive. The samples included a cross section, top, and bottom portion of the material. The micrographs were collected using the secondary electron imaging mode with an accelerating voltage of 5 kV, a spot size of 5.0, and a working distance of 10 mm. Images were processed in Python 3.9 via PIL and Matplotlib libraries to remove software artifacts and extract metadata from which the scale bars are produced. 

## 3. Results and Discussion

**Figure 4:**
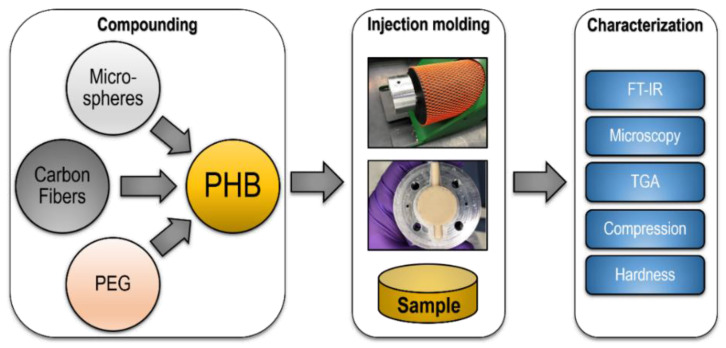
Sample process of preparing PHB based composites, through injection molding, to characterization.

The carbon fibers, microspheres, and PEG at concentrations ranging from 1–3%, 1–3%, and 5–25%, respectively, were separately combined with PHB granules in an extruder. The process involved the heating and compounding of the polymers and additives. Then, this mix was pushed through into the mold to form the test samples (Figure 4). The typical melting point for PHB is approximately 180 °C, and the initiation of decomposition is roughly above 220 °C [28]. Based on these properties, the material was processed at 200 °C to prevent degradation, while still having a fully melted polymer.

### 3.1. Microscopy Analysis

**Figure 5:**
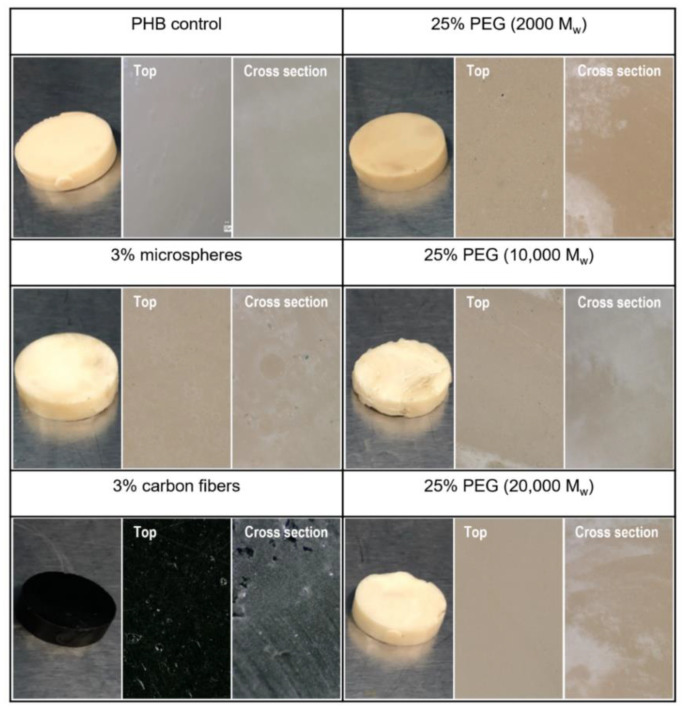
PHB composite sample surfaces photographed at 1×, 20×, and 100× for the analysis of surface changes. The 20× and 100× photographs were taken under full ring lighting conditions.

Figure 5 shows the color variations across the samples. The microsphere/PHB sample remained visually similar to the control sample. In the PEG/PHB composites, darker areas of brown were seen along the surface, most noticeably for the 2000 MW sample. This discoloration, could have been the result of the degradation of PHB or PEG; or incomplete mixing between the polymer and additives [29]. Since the PHB was processed below the degradation temperature (T_d_), the discoloration was likely due to the degradation of the PEG. In fact, previous studies have shown that PEG can start degrading at temperatures as low as 45 °C at relatively low MW values (6000) [30]. The PEG/PHB sample prepared with 20,000 MW PEG had a similar appearance with respect to the control sample. As expected, the carbon fiber/PHB composite showed a significant change in color from tan to black. Additional microscopy photographs can be found in Figure S1.

High-resolution confocal microscopy photographs highlight a fairly low number of distinguishable features from 500× to 2000×, as shown in Figure S2. The samples appear uniform, and no visible defect can be highlighted. The sample with 2000 MW PEG, as expected, shows a slight discoloration. The sample with PHB does show some porosity. However, at these resolutions, we can make no assessment of the distribution. High-definition images of the latter show that the pores are evenly distributed throughout the sample, as shown in Figure S3. Finally, SEM images of the same samples show samples that are uniform with no significant defects or multiphases to be distinguished, aside from the sample with microspheres that highlight well-dispersed pores ranging from a few microns to slightly over 100 µm, as shown in Figure S4.

**Figure 6:**
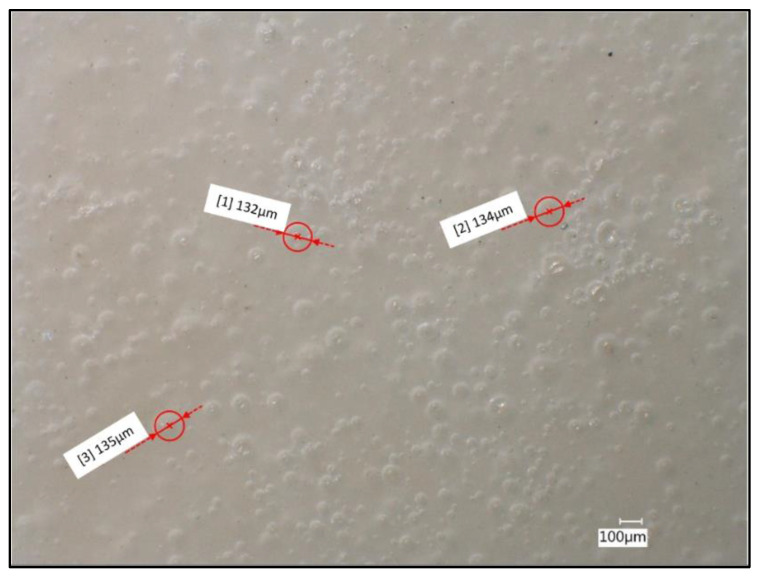
PHB/microsphere (3%) composite sample surface with measured pore diameters of fully formed microspheres. Image taken at 100× under partial-ring lighting conditions.

Using confocal microscopy and the measurement tool, it was possible to see the porosity change in the material from the expansion of the microspheres, as shown in Figure 6. As the spheres underwent processing in the extruder at high temperature, the thermal expansion of the microspheres was initiated. Based on the manufacturer specification, the microspheres reached approximately 120 µm in its expanded form. From the measurement of the pores, it was found that some of the microspheres expanded from 25 µm to approximately 134 µm. The remaining unexpanded spheres, were likely due to the inadequate time under room temperature during processing. Despite only a fraction of spheres reaching full expansion, the density of the samples was decreased.

We were able to determine a rough estimate of the density of our samples given the dimensions of the discs and their weight, as shown in Table 1. The use of microspheres was aimed at reducing the density of our material, offering a lighter material. As we increased the concentration of hollow fillers, the density decreased, similar to previous studies [31]. We noted that while the density did decrease, it was by much less than expected with a reduction of only 11.5%. This was possibly due to the increased pressure from the geometry of the die, as well as an incomplete expansion of the microspheres.

### 3.2. IR Spectroscopy Analysis

**Figure 7:**
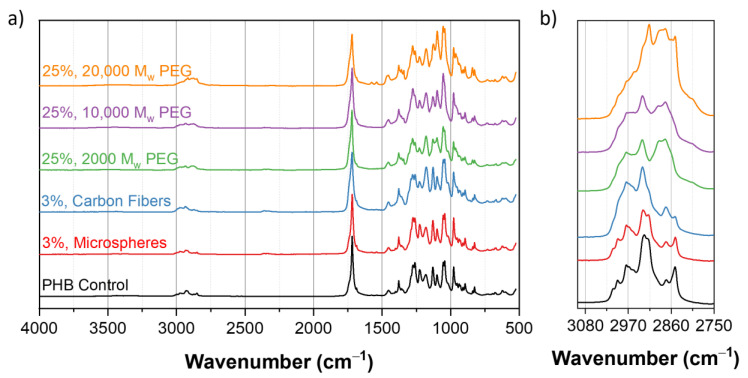
(**a**) IR spectra of PHB composites in the 500 to 4000 cm^−1^ frequency range. Absorption bands at 1280 and 1720 cm^−1^ are representative of the PHB content. (**b**) IR spectra in the 2750 to 3100 cm^−1^ range, for emphasizing on the effects of the PEG content at the 2930 cm^−1^ peak.

Infrared spectra of the PHB control and composites were collected, as shown in Figure 7. All of the spectra showed a strong absorption band at the 1720 and 1280 cm^−1^ peaks, corresponding to a C=O carbonyl group and C-O from the ester group, respectively, and are commonly observed when studying PHB [32]. The consistent presence of these peaks and similar peak ratios across the samples suggests that the PHB was mostly unchanged during the extrusion process. This absence of degradation phenomena is further confirmed, when compared against the IR spectra of the unprocessed PHB polymer (shown in Figure S6). The 2930 cm^−1^ peak corresponds to the alkane groups present in the PEG. Similarly, PHB has a presence of alkane groups at this peak. As a result, the groups for the two molecules overlap causing the peak to broaden [33]. Additional IR spectra that highlight different concentrations of additives can be found in Figure S5. 

### 3.3. Thermal Analysis

**Figure 8:**
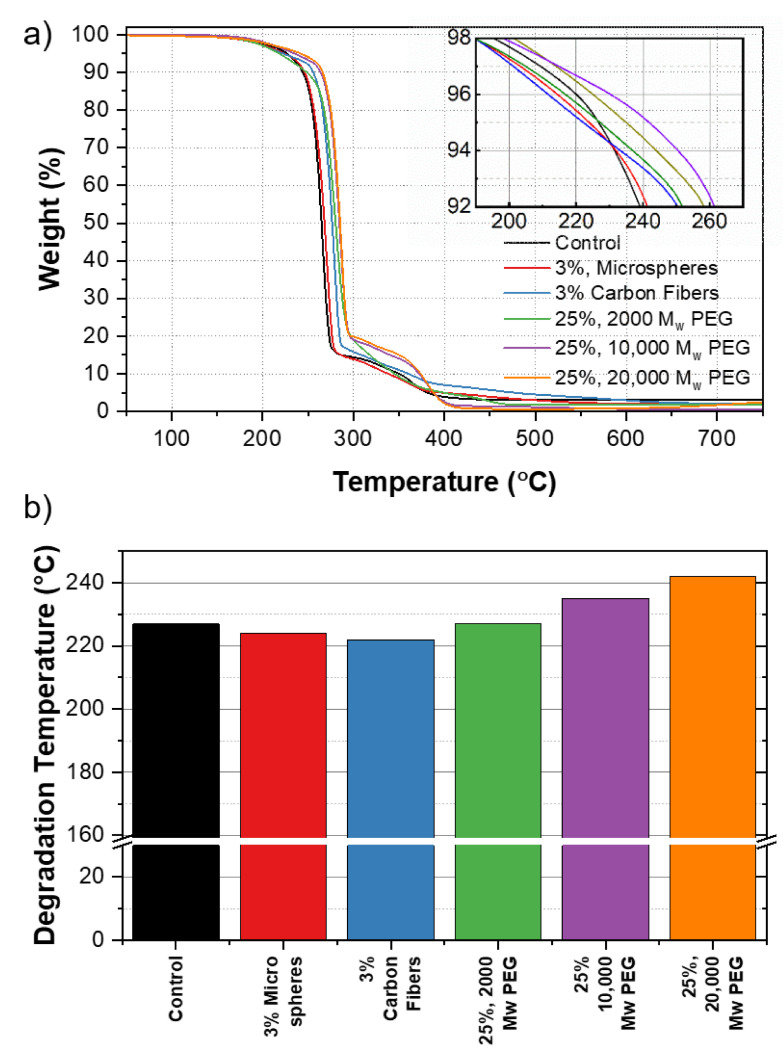
(**a**) TGA curves of PHB composites. The inset shows the degradation temperature, T_d_ across samples. (**b**) Comparison of T_d_ for PHB control against PHB composite samples.

Figure 8a shows the changes in degradation temperature across various samples. The T_d_ values reported correspond to a 5% weight loss in the material [34].

Figure 8b shows the baseline T_d_ value for PHB of 227 °C, corresponding to 5 wt% mass loss. The addition of 3% of the carbon fibers and microspheres shows a negligible change of 222 and 224 °C, respectively. The addition of 25% 2000, 10,000, and 20,000 MW PEG showed a change in T_d_ from 227 °C in the control samples without additives, to 227, 235, and 242 °C, respectively. As the MW of the PEG was increased, the thermal stability of the polymer was improved resulting in a higher temperature of degradation. We believe that this was due to the increasing length of the polymer chain, influencing the thermal stability of the material directly as MW increased [35]. Additional TGA curves for each additive at various concentrations can be found in Figure S7. 

### 3.4. Mechanical Characterization: Hardness

Material hardness is essential to understand how the material will resist plastic deformation and penetration. The enhancement of mechanical response of PHB composites through the compounding of additives could lead to new applications. 

**Figure 9:**
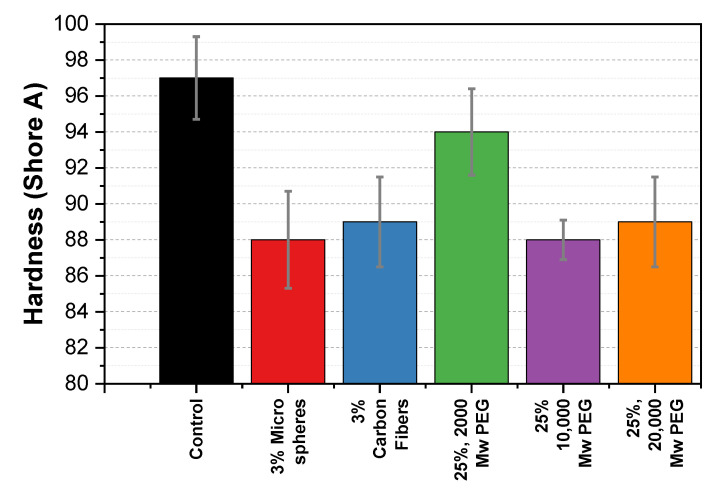
Shore A hardness comparison of PHB composite samples against the control sample. The bar shows the mean hardness (±standard deviation) of five measurements on each sample.

Figure 9 shows a Shore A value of 97 for the control sample. The addition of 3% carbon fibers and microspheres resulted in a decrease in Shore A values to 89 and 88, respectively. Similarly, the addition of 25% 2000, 10,000, and 20,000 MW PEG also reduced the hardness to 94, 88, and 89, respectively. 

The PHB composites in this study exhibited a decrease in hardness with the introduction of each of the additives. A study by Gavali et al. on polylactic acid (PLA)/carbon fiber composites reported an 85% increase in hardness after adding 20% (*w*/*w*) carbon fibers [36]. Therefore, we expected to see a similar increase. However, as seen in Figure 9, we found a decrease in the hardness for the carbon fiber sample. In this paper, we propose that the carbon fibers disrupt the intramolecular and intermolecular interactions (hydrogen bonding and Van der Waals) of the PHB chains and therefore, weaken the lattice structure, making the samples softer. In addition, we extend this explanation to the microspheres sample. 

PEG is often used as a plasticizer for lowering the rigidity of materials. We anticipated that this effect would lower the hardness of the composite. We found that the PEG did in fact lower the hardness of the material for the 10,000 and 20,000 MW samples. At higher molecular weights, we believe the PEG acts as an effective plasticizer by allowing the PHB chains to slide past each other when a force is applied. Additional hardness figures for each additive at various concentrations can be found in Figure S8.

### 3.5. Mechanical Characterization: Compressibility

Compression tests are used to understand the material performance under loading conditions, ductility, and compressibility. As we apply a load to the sample, we can further explore how the material will deform/displace. 

**Figure 10:**
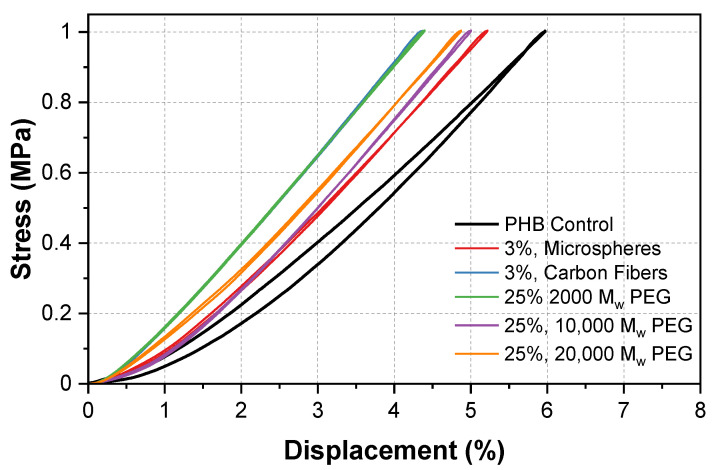
Stress/displacement diagram of PHB composites under 1 MPa compression for the analysis of maximum displacement.

Figure 10 shows a displacement of 6% for our control sample. The addition of 3% of the carbon fibers and microspheres showed a lowered displacement of 4.4 and 5.2%, respectively. Similarly, the addition of 25% 2000, 10,000, and 20,000 MW PEG showed a lowered displacement of 4.5, and 5%, respectively. Of note, all of the values have an experimental error of ±0.15% due to the instrument.

The PHB/microsphere composite showed a decrease in displacement from 6 to 5.2%. In previous work on macromolecular microsphere composite hydrogels, an increase in displacement was seen as a result of the microspheres [37]. This was opposite to our composite. Here, we suggest that the microsphere and carbon fiber additives occupied free space in the polymer matrix. As the samples were compressed, the chains were no longer able to rearrange as freely, which resulted in a decrease in the displacement. Unlike in the hardness tests that applied a force to a specific point, the compression tests applied a force to the entire sample creating a closed system that limited the mobility of the polymer chains.

A decrease in displacement was seen across all of the PEG/PHB composites. The compression properties of the composites depend on the polymer (PEG) chain length and how the material was prepared. At low densities and compression rates, a solid-like behavior occurs, which is most common in shorter chains. This could explain why the lowest displacement occurred at a MW of 2000. As the MW of the PEG in the sample was increased, the compressibility also increased, due to the longer chain length. This process could be interpreted as a polymer chain rearrangement, which develops in slow compression rates [38]. Additional stress/displacement curves can be found in Figure S9. In addition, a bar graph highlighting the changes in maximum displacement for the highest additive concentrations is shown in Figure S10.

The experiments conducted in this study were necessary to gain a baseline understanding of the effects of the selected additives on PHB. First, using both high-resolution confocal and SEM microscopy to analyze the morphology of the different PHB samples produced, we determined that despite the fact that only a single screw extruder was used to perform the compounding, which introduced the various additives into the polymer matrix, the samples appeared uniform, with no apparent segregation into multiple phases. The PHB sample with low MW PEG shows a slight discoloration/browning of the material, likely due to a premature degradation. Microscopy also highlighted an incomplete expansion in some of the microspheres that could stem from a pressure increase in the mold during processing. Second, IR spectroscopy has shown that PHB was mostly unaltered during the extrusion process. This phenomenon was further confirmed through a comparison between the composites and the IR spectra of the unprocessed PHB polymer (Figure S6). In addition, we found that the thermal stability of PHB/PEG composites was improved with higher molecular weights, possibly due to the interaction of the polymer chain with the PEG. Finally, mechanical testing showed a decrease in hardness, as well as a decrease in compressibility for all of the samples. The proposed mechanisms that could help explain these results are the intra- and intermolecular interactions from the carbon fibers, the reduced density from the microspheres, and the plasticizing effects of the PEG that overall disrupt the crystallinity of the material. Furthermore, the occupied free space of the microspheres and carbon fibers, as well as the solid-like behavior from the PEG could explain the unexpected lack of flexibility of these materials.

Compared to other bioplastics, such as PLA, PHB exhibits a lower thermal stability and mechanical properties that make it less desirable for mass market applications [39]. While PLA-PHB blends are promising optiona for the replacement of traditional plastics, focusing on PHB-only polymers is crucial for end-of-life considerations, such as in single use plastics [10]. Improving on the degradation temperature and expanding on the process temperature range, as was done in this study, is essential for broadening the single use applications of these materials. For instance, this allows the consideration of PHBs not only for single use packaging, such as cups, plates, and utensils, but also for the expansion to more challenging applications, such as traditional or microwave oven meals and hot beverages [40]. 

## 4. Conclusions

This work served as a preliminary study of the effect of carbon fibers, microspheres, PHB, and PEG on PHB composites, and contributed to understanding the thermal and mechanical behaviors of these materials. We compounded PHBs with various concentrations of additives using a single-screw extruder retrofitted with an in-house injection mold. The injection-molded samples were an attempt to use a state-of-the-art industrial technique to produce the parts. We investigated the changes of properties, which are induced by the addition of carbon fibers, microspheres or PEG additives using microscopy, spectroscopic, thermal, and mechanical techniques. We have shown that the PEG of higher MW had an improved thermal stability, slightly increasing the T_d_ of these composites. In addition, we noted that the percentage displacement of the PEG composites was slightly higher at higher MWs.

The addition of microspheres did not have noticeable effects on T_d_, presumably due to an incomplete expansion of the microspheres. Both the carbon fibers and microsphere additives resulted in a decreased hardness of the composites, likely from the disruption of the interactions between the PHB chains. In this study, the PHB and additives were mixed under a relatively short mixing time in the extruder, as they underwent a single pass. If the material had been processed continuously, as in an industrial complex, we may have obtained a more homogenous mixture. While the effects of the additives used in this study were subtle, the results were repeatable. In future studies, additional aspects of injection molding processes may be adopted, in order to investigate the effects of injection molding beyond utilization as a sample formation method. Our results help in building a foundation for systematic composite manufacturing and characterization that will enable novel applications for PHBs and a wider adoption across industries. With single-use plastics contributing heavily towards global plastic pollution, the design of viable bioplastic composites will allow the development of new applications for these promising materials. Expanding the thermal and mechanical capabilities of biopolymer composites to reach higher temperatures and levels of mechanical stress/strain, will increase the viability of the materials as replacements for traditional non-biodegradable polymers. 

With the improvements explored in this study, we continue to expand the possibilities of PHB composites. As the T_d_ is improved further, more demanding temperature applications, such as microwavable plastics, hot beverage products, and high temperature packaging are increasingly achievable. Similarly, as additional improvements are made on the mechanical properties of PHB, the applications in foams, appliance packaging, and electronic packaging are likely candidates. Future studies on the oxygen diffusion, water barrier, and elasticity properties of PHB will continue to expand the applications of this polymer. 

## Figures and Tables

**Table 1:** Density of PHB/microsphere composites.

Name	Density (g/cm^2^)
PHB (control)	1.05
1% Microspheres	1.04
2% Microspheres	0.95
3% Microspheres	0.94

## Data Availability

All of the data needed to evaluate the conclusions in the paper are present in the paper and/or the Supplementary Materials. Additional data related to this paper may be requested from the authors.

## Supplementary Materials

The following are available online at https://www.mdpi.com/article/10.3390/polym13244444/s1, Figure S1: PHB composite sample surface at 100× under full ring lighting, Figure S2: IR Spectra for PHB composite samples, Figure S3: TGA curves for PHB composites, Figure S4: Shore A hardness for PHB composites, Figure S5: Stress/strain diagrams for PHB composites, Figure S6: Maximum displacement diagram for PHB composites, Figure S7:TGA curves for PHB composites, Figure S8: Shore A hardness for PHB composites, Figure S9: Stress/strain diagrams for PHB composites, Figure S10: Maximum displacement diagram for PHB composites.
