# Supplementary material for: Characterization of Polyhydroxybutyrate-Based Composites Prepared by Injection Molding
Source: Polymers (Basel). 2021 Dec 18;13(24):4444. doi: 10.3390/polym13244444 (PMC8704503; doi:10.3390/polym13244444)
Supplement: Supplementary file 1 [file polymers-13-04444-s001.zip › polymers-1469945-supplementary.pdf]

polymers

MDPI

Supplementary Information

Characterization of Polyhydroxybutyrate-Based Composites Prepared by Injection Molding

Marcos M. Hernandez, Nevin S. Gupta, Kwan-Soo Lee, Aaron C. Pital, Babetta L. Marrone, Carl N. Iverson and Joseph H. Dumont *

Los Alamos National Laboratory, Los Alamos, NM 87545, USA; mhernandez@lanl.gov (M.M.H.); nevin5@lanl.gov (N.S.G.); kslee@lanl.gov (K.-S.L.); acpital@lanl.gov (A.C.P); blm@lanl.gov (B.L.M.); iverson@lanl.gov (C.N.I.)

* Correspondence: joseph.dumont@lanl.gov

Citation: Hernandez, M.M.; Gupta, N.S.; Lee, K.-S.; Pital, A.C.; Marrone, B.L.; Iverson, C.N. Characterization of Polyhydroxybutyrate-Based Composites Prepared by Injection Molding. *Polymers* **2021**, *13*, 4444. https://doi.org/10.3390/polym13244444

Academic Editors: Domenico Acierno and Antonella Patti

Received: 2 November 2021
Accepted: 13 December 2021
Published: 18 December 2021

Publisher's Note: MDPI stays neutral with regard to jurisdictional claims in published maps and institutional affiliations.

Copyright: © 2021 by the authors. Licensee MDPI, Basel, Switzerland. This article is an open access article distributed under the terms and conditions of the Creative Commons Attribution (CC BY) license (https://creativecommons.org/licenses/by/4.0/).

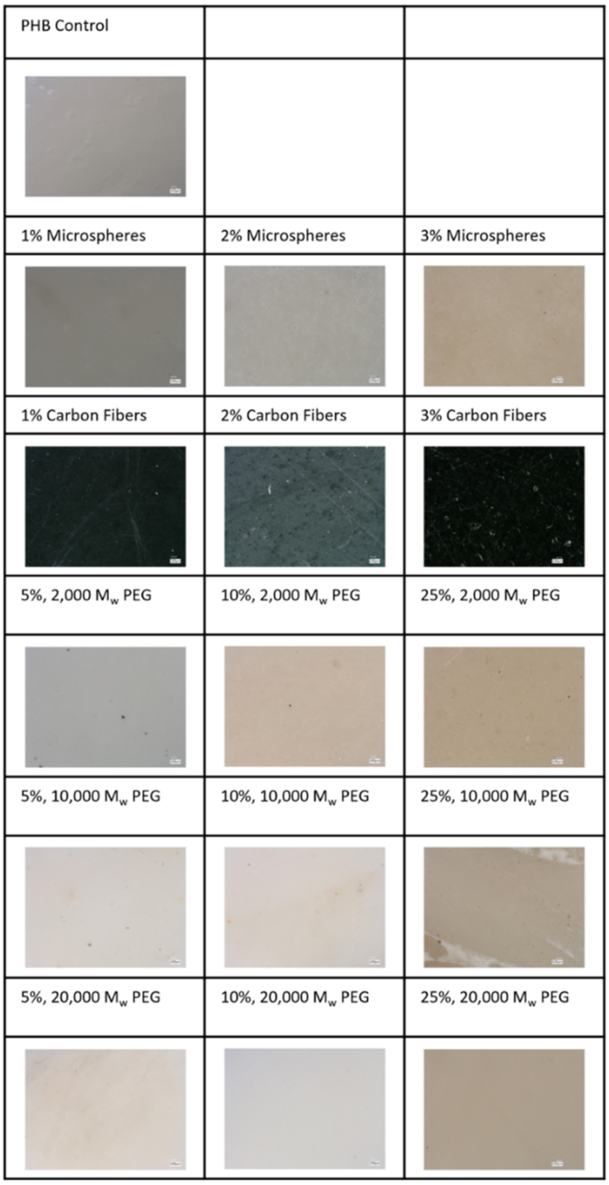

Figure S1. PHB composite sample surface at 100x under full ring lighting.

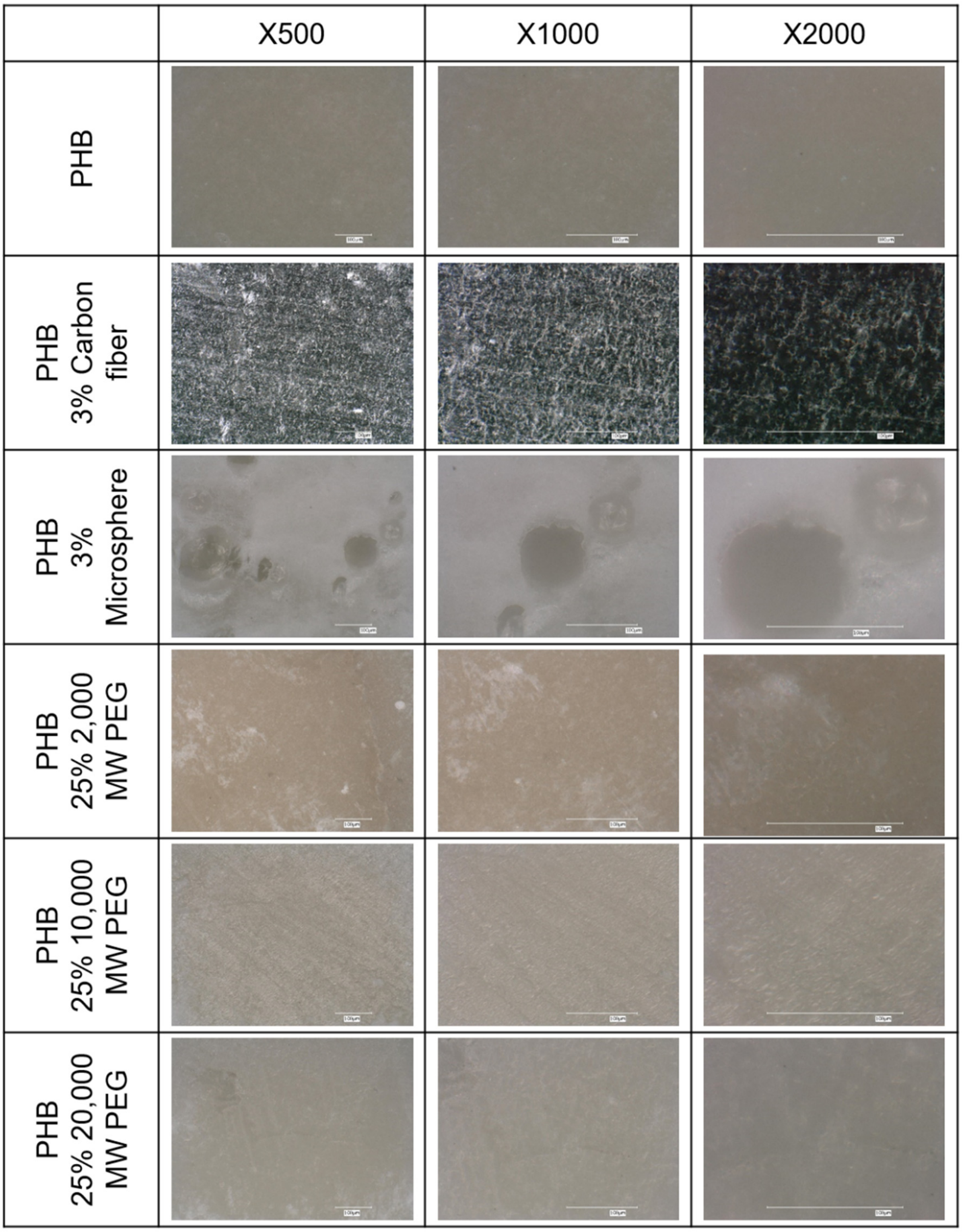

Figure S2. High resolution confocal photographs of PHB composite samples at 500x, 1000x, and 2,000x

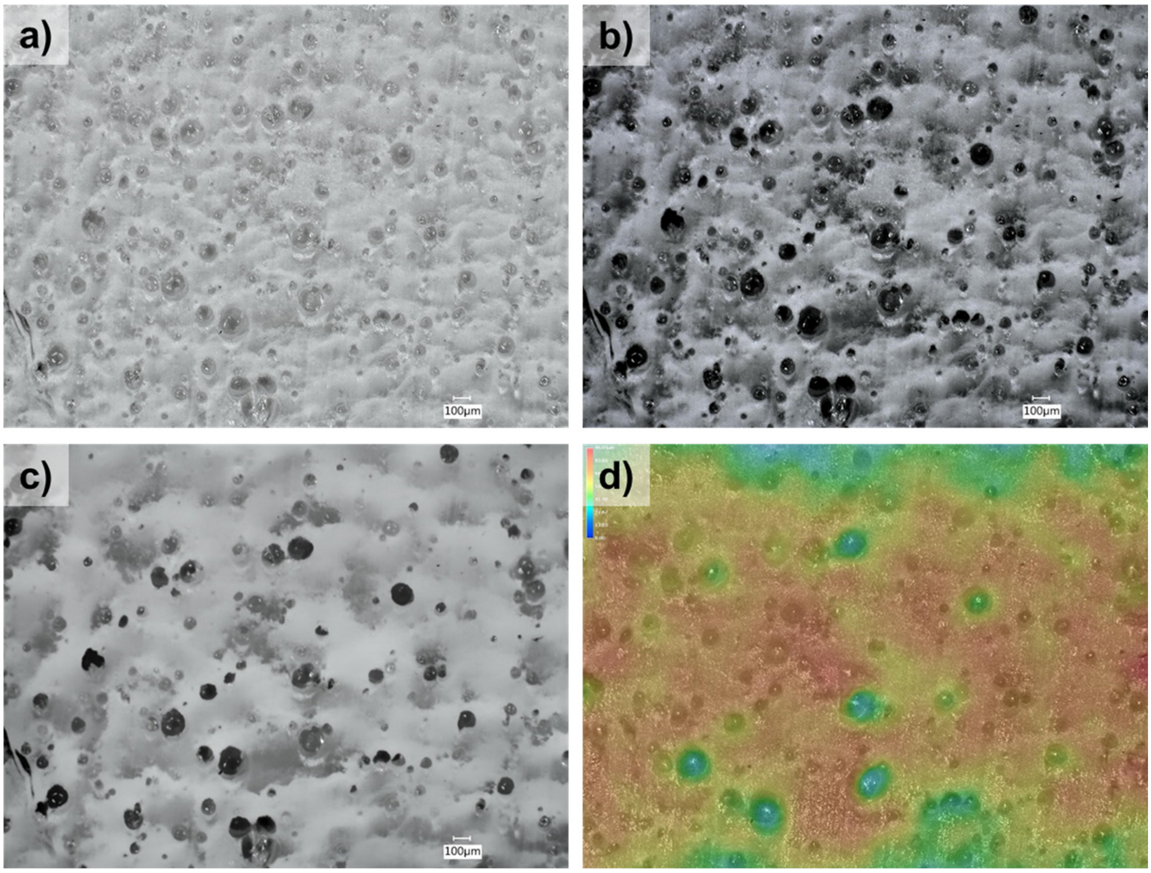

Figure S3. Surface morphology photographs of 3% microsphere/PHB composites at 100x. a) No treatment to the image, b) High definition, with increased contrast, c) High definition, with increased contrast, and smooth surface and d) 3D rendering of image b)

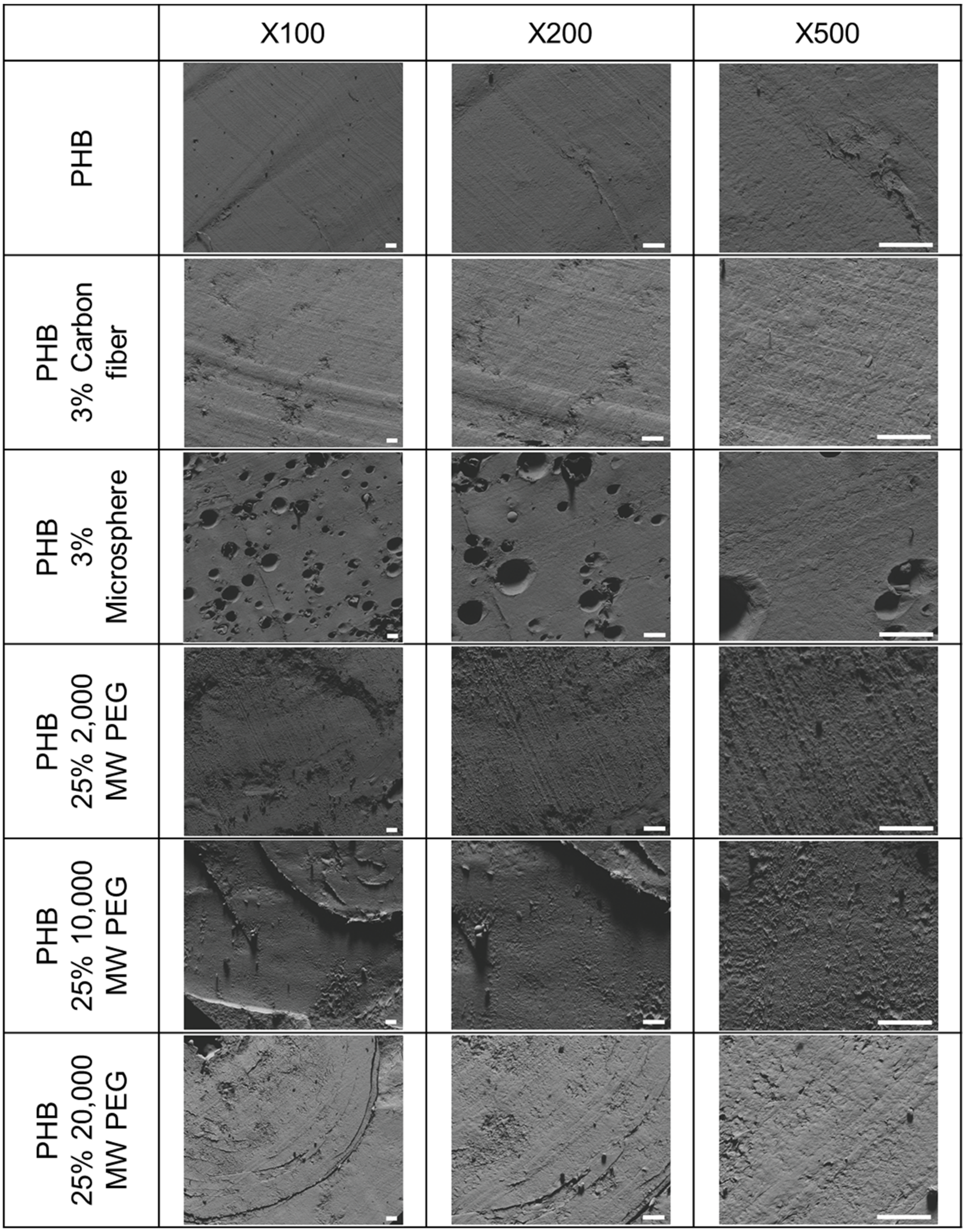

Figure S4. SEM images of PHB samples prepared by single screw extruder. The scale bar represents 100 μm.

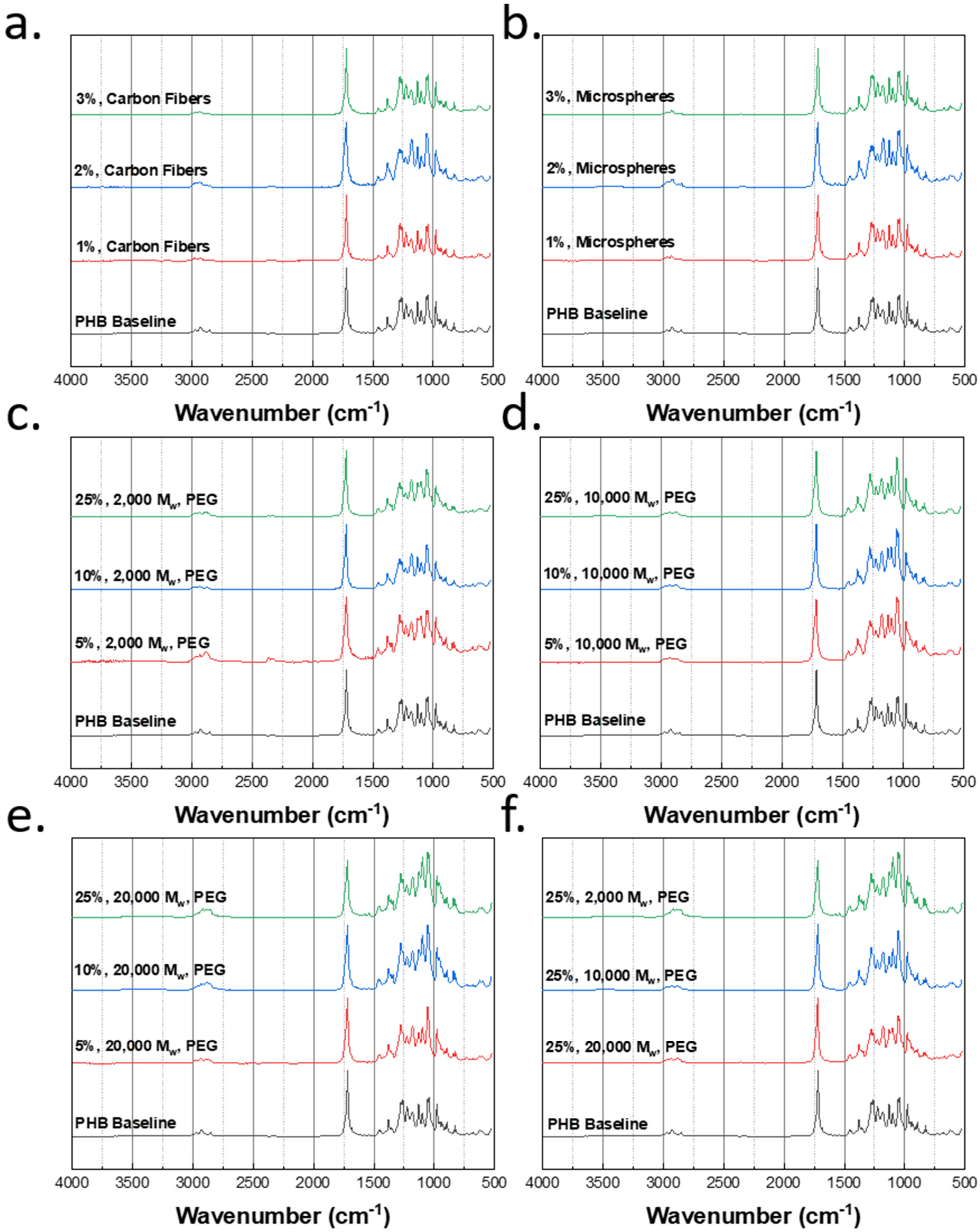

Figure S5. IR Spectra for PHB composite samples

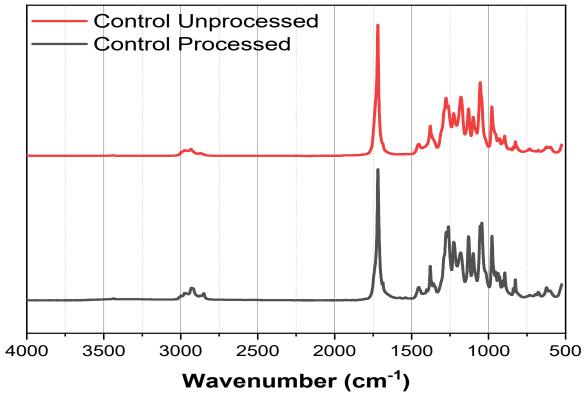

Figure S6. FTIR spectra of both processed and unprocessed PHB.

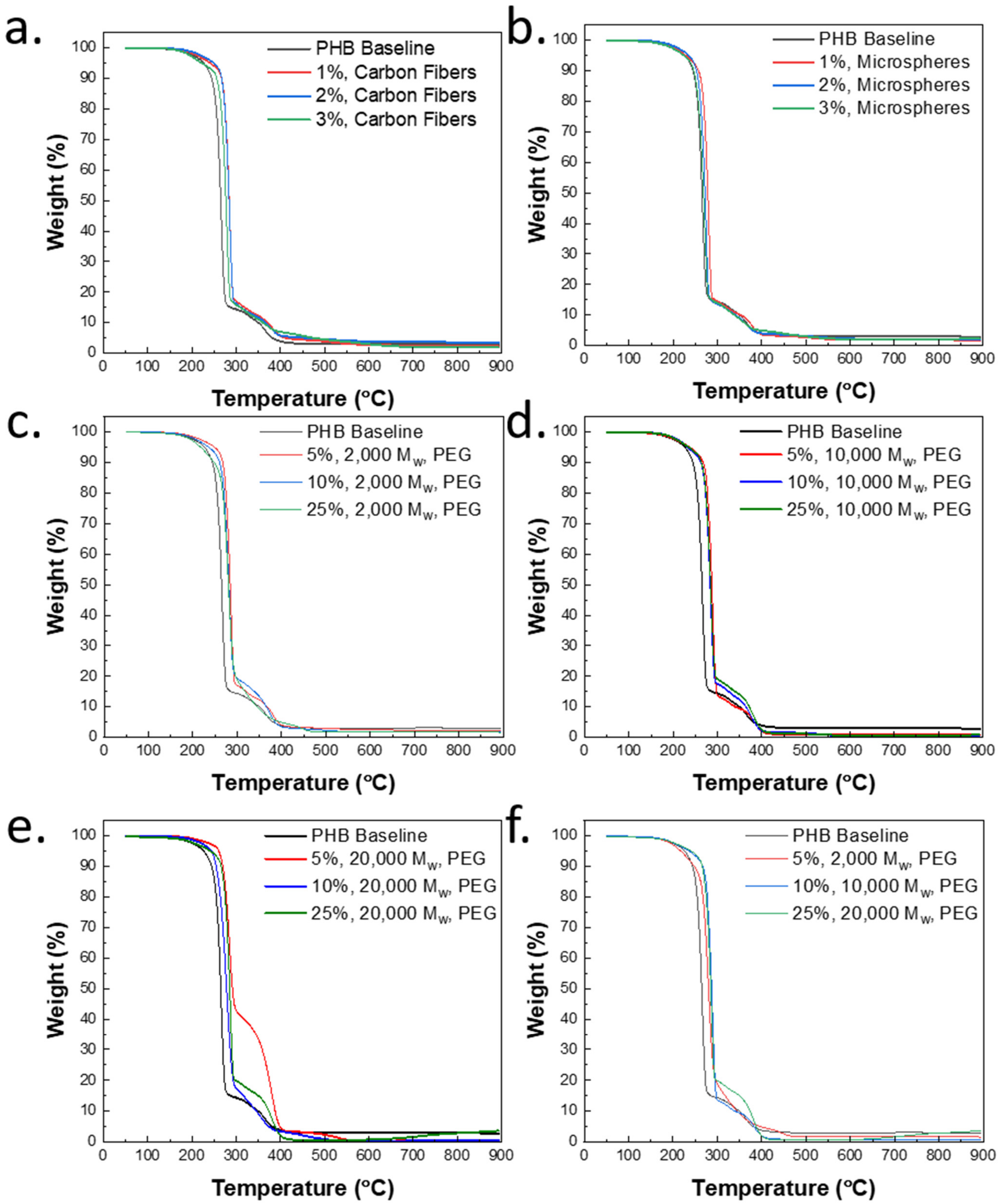

Figure S7. TGA curves for PHB composites.

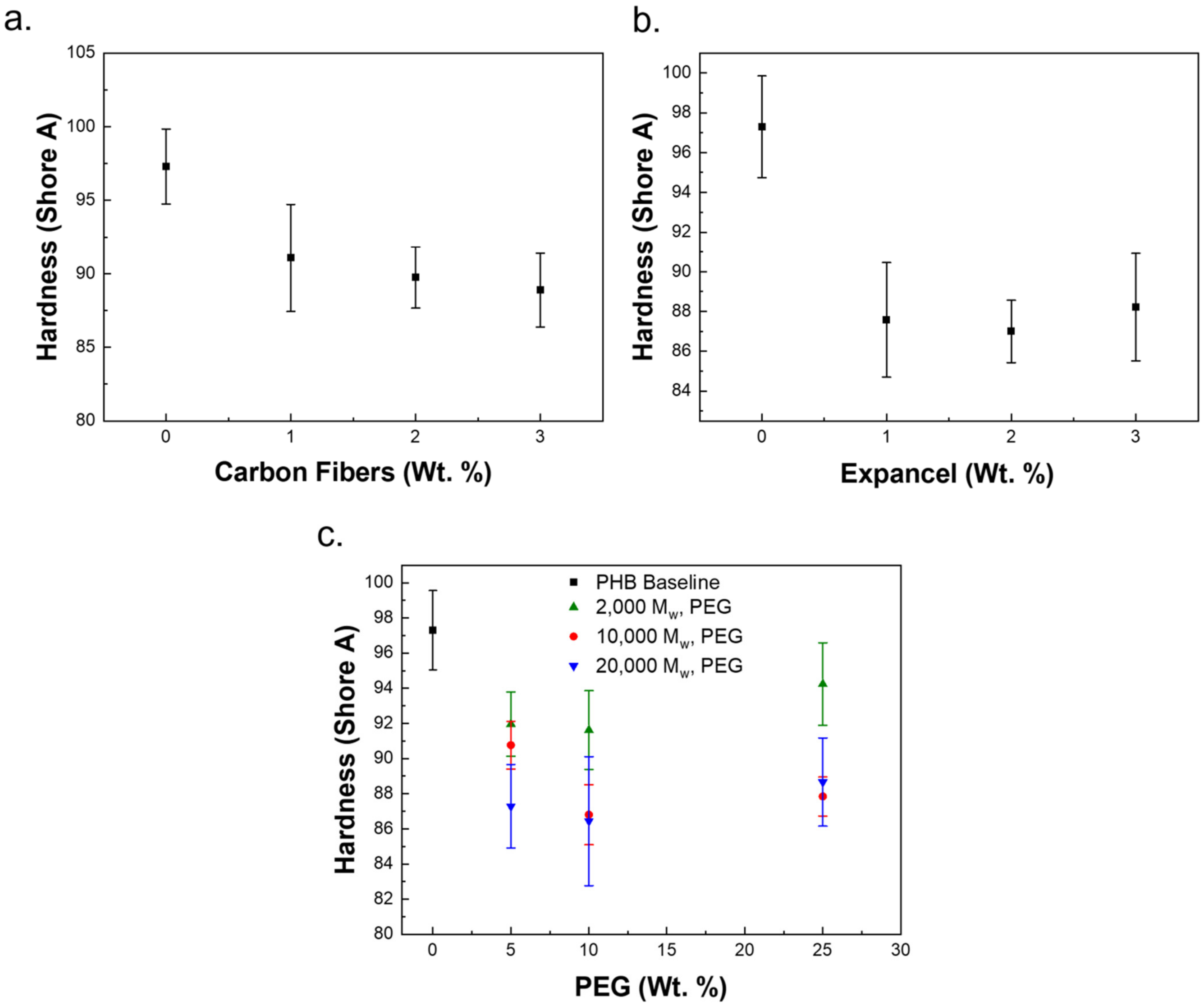

Figure S8. Shore A hardness for PHB composites

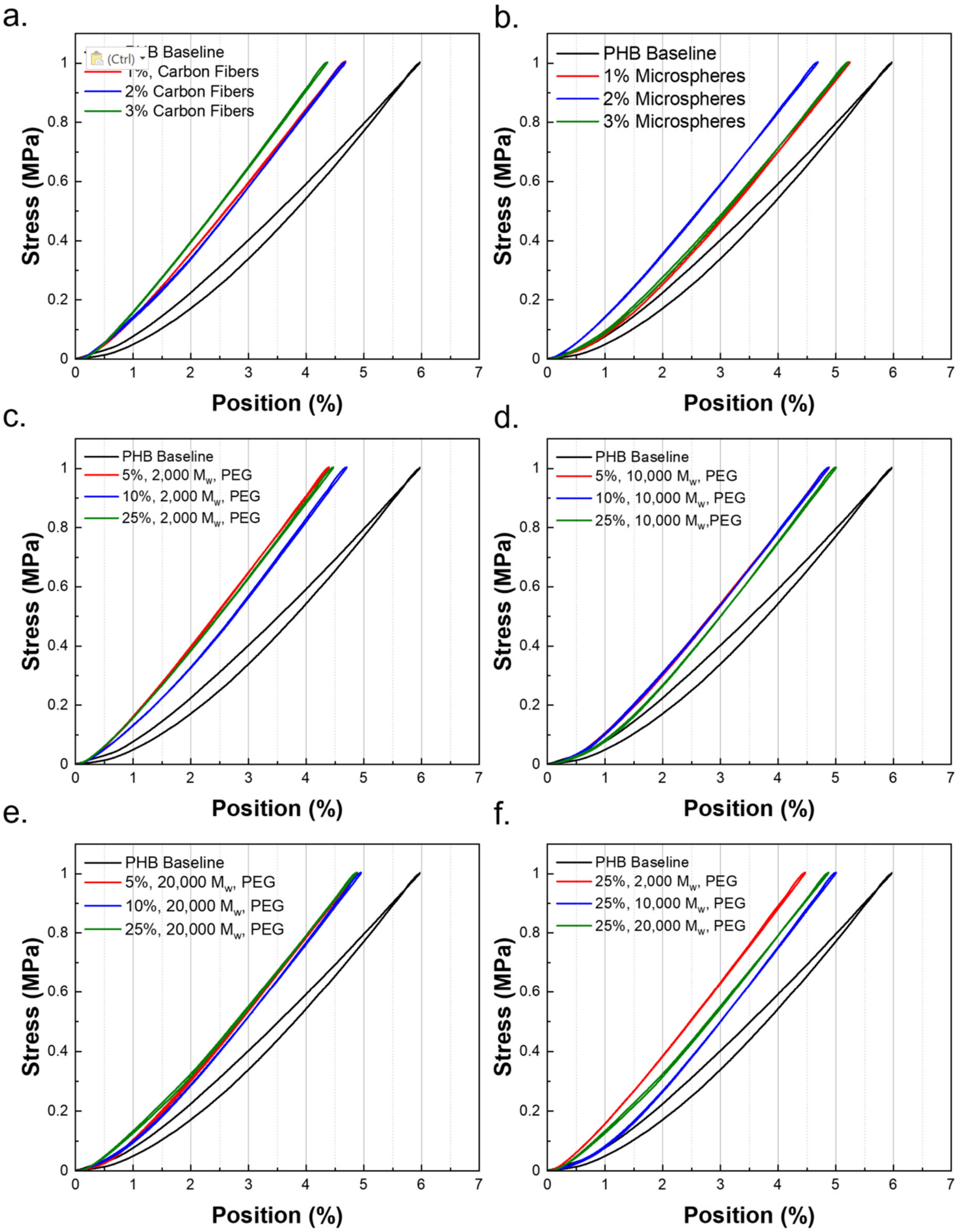

Figure S9. Stress/strain diagrams for PHB composites

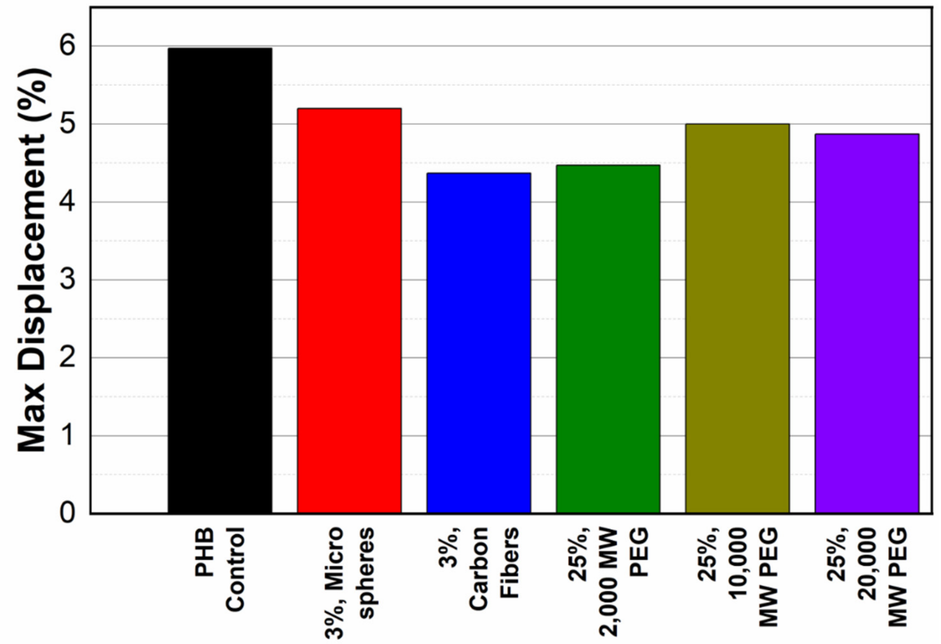

Figure S10. Maximum displacement diagram for PHB composites
